# A Gaussian Mixture Model-Based Unsupervised Dendritic Artificial Visual System for Motion Direction Detection

**DOI:** 10.3390/biomimetics10050332

**Published:** 2025-05-19

**Authors:** Zhiyu Qiu, Yuxiao Hua, Tianqi Chen, Yuki Todo, Zheng Tang, Delai Qiu, Chunping Chu

**Affiliations:** 1 Division of Electrical, Engineering and Computer Science, Graduate School of Natural Science & Technology, Kanazawa University, Kakuma, Kanazawa 920-1192, Japan; qiuzy1916@stu.kanazawa-u.ac.jp (Z.Q.); sh13818971028@gmail.com (Y.H.); chentianqi@stu.kanazawa-u.ac.jp (T.C.); 2 Faculty of Electrical and Computer Engineering, Kanazawa University, Kakuma-Machi, Kanazawa 920-1192, Japan; 3 Institute of AI for Industries, Chinese Academy of Sciences, 168 Tianquan Road, Nanjing 211135, China; 4 Brain Science Institute, Jilin Medical University, Jilin 132000, China; dlqiu@jlmu.edu.cn (D.Q.); cpchu@jlmu.edu.cn (C.C.)

**Keywords:** artificial visual system, unsupervised learning, neuron, motion direction detection, GMM

## Abstract

Motion perception is a fundamental function of biological visual systems, enabling organisms to navigate dynamic environments, detect threats, and track moving objects. Inspired by the mechanisms of biological motion processing, we propose an Unsupervised Artificial Visual System for motion direction detection. Unlike traditional supervised learning approaches, our model employs unsupervised learning to classify local motion direction detection neurons and group those with similar directional preferences to form macroscopic motion direction detection neurons. The activation of these neurons is proportional to the received input, and the neuron with the highest activation determines the macroscopic motion direction of the object. The proposed system consists of two layers: a local motion direction detection layer and an unsupervised global motion direction detection layer. For local motion detection, we adopt the Local Motion Detection Neuron (LMDN) model proposed in our previous work, which detects motion in eight different directions. The outputs of these neurons serve as inputs to the global motion direction detection layer, which employs a Gaussian Mixture Model (GMM) for unsupervised clustering. GMM, a probabilistic clustering method, effectively classifies local motion detection neurons according to their preferred directions, aligning with biological principles of sensory adaptation and probabilistic neural processing. Through repeated exposure to motion stimuli, our model self-organizes to detect macroscopic motion direction without the need for labeled data. Experimental results demonstrate that the GMM-based global motion detection layer successfully classifies motion direction signals, forming structured motion representations akin to biological visual systems. Furthermore, the system achieves motion direction detection accuracy comparable to previous supervised models while offering a more biologically plausible mechanism. This work highlights the potential of unsupervised learning in artificial vision and contributes to the development of adaptive motion perception models inspired by neural computation.

## 1. Introduction

Motion perception is a fundamental capability of biological visual systems, essential for survival and effective interaction with ever-changing environments. Over millions of years of evolution, these systems have developed highly efficient and specialized mechanisms for detecting and processing motion direction, enabling organisms to respond rapidly to potential threats, locate prey, and navigate complex surroundings [1,2,3,4,5,6]. In recent years, the study of motion vision has gained significant attention, not only due to its relevance in advancing image-processing technologies but also because it provides valuable insights into the underlying neural computations of the brain [7,8,9,10]. Understanding how biological systems perceive motion can inspire the development of more sophisticated artificial vision models and improve real-time motion detection in various applications [11,12].

Traditional machine learning approaches for computer vision often depend on supervised learning, requiring large, labeled datasets and extensive computational resources [13,14]. Although supervised learning models have achieved remarkable success across various domains, researchers have pointed out that continuous mathematical optimizations in modern deep learning models have led to a growing divergence from the mechanisms of the human brain [15,16]. Moreover, studies suggest that biological visual systems do not depend on labeled data; instead, they leverage unsupervised processes that self-organize through repeated exposure to motion stimuli [17,18,19]. This natural adaptation enables the formation of structured and highly efficient neural representations. Recent studies further support this view by demonstrating biologically plausible unsupervised learning frameworks that can capture complex visual dynamics without labeled supervision [19,20,21]. For example, Ligeralde et al. [19] showed that efficient spatial representations can emerge in neural networks solely through training on spontaneous retinal activity, aligning with early-stage biological development. Studies have also demonstrated that motion selectivity, including direction and speed, can emerge in an unsupervised manner within hierarchical spiking neural networks. For instance, Paredes-Vallés et al. [22] presented a spiking architecture where motion selectivity developed through unsupervised learning from raw stimuli captured by event-based cameras. Additionally, perceptual learning studies have shown that training on motion direction discrimination tasks can enhance sensitivity to motion through unsupervised learning processes. For example, research by Thompson et al. [23] demonstrated that participants improved their ability to discriminate motion direction after repeated exposure to motion stimuli, suggesting that the visual system can adapt to motion information without explicit supervision.

The processing of visual motion begins in the retina, where specialized retinal ganglion cells, particularly direction-selective ganglion cells (DSGCs), respond to motion stimuli and encode initial directional information [24]. These signals are then transmitted via the optic nerve to the lateral geniculate nucleus (LGN) of the thalamus, which serves as a relay station before forwarding visual input to the primary visual cortex (V1) [25]. Within V1, motion information is further processed by orientation-selective and motion-sensitive neurons, which extract essential motion features such as direction and speed. From there, the signals are sent to the middle temporal (MT) and medial superior temporal (MST) areas, where neurons become highly selective to motion direction and complex motion patterns [26,27]. These areas undergo synaptic plasticity and self-organizing processes, allowing for continuous adaptation and refinement of motion perception [28,29]. Studies on unsupervised learning in biological vision have shown that neurons can develop motion selectivity purely through exposure to motion stimuli, without requiring explicit feedback or labeled training data [12,30].

In our previous research, we constructed various motion direction-detecting models inspired by the visual system [31,32,33]. Most studies focused on modeling direction-selective cells in the retina, forming local motion direction-detecting models. For global motion direction detection, since most models are built based on the visual systems of adult individuals, we merely assumed that the outputs of motion direction-selective neurons with the same preferred direction converge, without detailed discussion, including the process of formation. Some studies have suggested that the number of activated motion direction-selective neurons in different directions influences the final perception of an object’s overall motion direction [34]. However, this phenomenon may develop through postnatal learning, and the precise mechanism by which local motion information integrates into global motion perception remains unclear.

In this paper, inspired by these principles, we propose a novel mechanism for detecting macroscopic motion direction. We performed unsupervised learning classification on local motion direction detection neurons, grouping the outputs of neurons detecting the same direction to form macroscopic motion direction detection neurons. The output of these macroscopic motion direction detection neurons is positively correlated with the received input. Ultimately, the neuron with the highest output represents the macroscopic motion direction of the object. Based on these principles, we propose an Unsupervised Artificial Visual System for motion direction detection. This model consists of two layers: the local motion direction-detecting neuron layer and the unsupervised learning global motion direction detection layer. In the local motion direction detection neuron layer, we adopt the Local Motion Detection Neuron (LMDN) model proposed by Hua et al. [35]. This model can detect motion in eight directions as well as speed. In this study, we use it to detect local motion directions. We used the modified output of the local motion direction-detecting neurons constructed in our previous work as the input to the global motion direction-detecting layer based on the Gaussian Mixture Model (GMM). In the global motion direction detection, we used the GMM to classify local motion detection neurons according to their preferred directions. The GMM is a widely used unsupervised learning method for clustering and density estimation. GMM represents complex data distributions as a weighted sum of multiple Gaussian components, capturing underlying patterns in high-dimensional data [36]. This probabilistic framework aligns with biological sensory processing, where neurons respond to stimuli with varying degrees of activation, akin to the overlapping distributions of Gaussian components. In the visual system, neurons in regions such as V1 and MT exhibit selective tuning to motion direction and spatial features, forming structured representations through continuous adaptation to sensory inputs [37]. Similarly, GMMs assign data points to different Gaussian components based on probability distributions, dynamically adapting to data structures. Unlike deterministic clustering methods, GMMs accommodate uncertainty and overlapping categories, reflecting the probabilistic nature of neural processing [38]. This alignment makes GMM a valuable tool for modeling biological motion perception and developing artificial visual systems that adaptively cluster the outputs of similar neurons together. At the end of the unsupervised learning layer, we selected the neuron with the maximum output as the global motion direction of the object, based on the theory of Cafaro et al. [34]. We demonstrate how this approach captures key properties of biological motion perception and enables the formation of a global motion direction-detecting system through repeated exposure to motion stimuli, providing a more natural method for motion detection in artificial visual systems. The results demonstrate that the Gaussian Mixture Model-based global motion direction-detecting layer can accurately classify signals from different directions after repeated exposure to motion stimuli from those directions.

## 2. Materials and Methods

### 2.1. Dendritic Neuron Model for Motion Direction Detection

In our previous work, we constructed multiple AVSs (Artificial Visual Systems) designed to detect motion in eight different directions [31,32,33]. The structure of these systems consists of two main components: the local motion direction detection layer and the global motion direction detection layer.

Our model typically uses two images as input, representing the scene before and after the motion occurs. The input images are first processed by the local motion direction detection layer, which corresponds to the retinal stage of the visual pathway. The number of nodes in this layer corresponds to the resolution of the input image, and each node is associated with eight types of neurons, each specialized in detecting motion in a different direction. These neurons detect the motion direction at the pixel level. Extensive research studies have shown that some retinal ganglion cells in the retina of mammals and certain neurons in the optic lobe of flies exhibit direction selectivity, meaning that they become activated only when stimulated by motion in a specific direction [2]. In our previous models, the local motion direction detection layers were primarily based on either Barlow’s model, the Hassenstein–Reichardt Correlator (HRC) model, or the dendritic neuron model [39,40,41]. Moreover, previous studies have demonstrated that these neuronal models can effectively extract local motion direction from two consecutive image frames. Although these models represent different fundamental approaches to motion detection in biological visual systems, they ultimately provide two key pieces of information: the detected motion direction and whether the neuron is activated. These two pieces of information serve as the input to the global motion direction detection layer, which corresponds to some parts of the visual cortex. Studies have shown that certain neurons in the visual cortex determine the overall motion direction by statistically analyzing the number of activated local motion direction-detecting neurons [34]. In previous models, neurons in the global motion direction detection layer count the activations of local motion direction neurons for each of the eight directions and determine the global motion direction based on the direction with the highest activation count.

**Figure 1:**
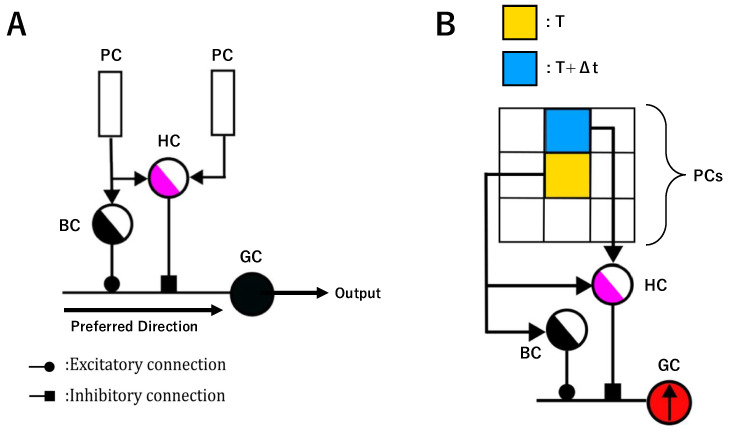
(**A**) Basic structure of the Local Motion Detection Neuron (LMDN) [35]. The model consists of photoreceptor cells (PCs), bipolar cells (BCs), horizontal cells (HCs), and ganglion cells (GCs). (**B**) Structure of the LMDN detects upward motion.

In this study, we adopt the Local Motion Detection Neuron (LMDN) model proposed by Hua et al. [35] for local motion direction detection. The LMDN simulates direction-selective ganglion cells (DSGCs) in the retina and detects motion across 8 directions (e.g., upper-right, leftward) and it is proposed based on the principle of the dendritic neuron model. Our decision to adopt this model rather than the HRC or Barlow models is supported by evidence suggesting that the dendritic neuron model provides a closer approximation to the functional characteristics of real biological neurons [42,43,44]. Figure 1A shows the structure of the LMDN. It consists of photoreceptor cells (PCs), bipolar cells (BCs), horizontal cells (HCs), and ganglion cells (GCs). Each LMDN type shares a hierarchical structure inspired by retinal layers and processes motion scenarios by scanning receptive fields with predefined spatial–temporal parameters.

Photoreceptor cells (PCs) convert light signals into electrical impulses. They are categorized into luminance-sensitive cone cells (CCs) and color-specific rod cells (L-RC, M-RC, S-RC) for RGB processing. Mathematically, their function is defined as $P_{i,j,t}=X$, where *X* represents the luminance value at coordinates (i,j) and time *t*. PCs route data to subsequent layers based on image type (grayscale or color), mimicking biological phototransduction.

Horizontal cells (HCs) detect edges by comparing luminance differences between neighboring regions. The lateral inhibition from horizontal cells enhances contour detection, critical for motion direction analysis. Using Equation (1), they activate when the absolute difference between a pixel’s luminance at time *t* and its neighbor at t+Δt exceeds a threshold *L*:

(1) $$HC=\left\{\begin{array}{cc} 1, & |X_{i,j,t}-X_{i+\alpha ,j+\beta ,t+\Delta t}\;|>L;\\ 0, & otherwise. \end{array}\right.$$

Bipolar cells (BCs) detect temporal changes by comparing sequential frames. They relay motion-triggered signals to ganglion cells, filtering static or insignificant luminance variations. Their activation logic (Equation (2)) mirrors HCs but focuses on time intervals:

(2) $$BC=\left\{\begin{array}{cc} 1, & |X_{i,j,t}-X_{i,j,t+\Delta t}\;|>L;\\ 0, & otherwise. \end{array}\right.$$

Ganglion cells (GCs) integrate synaptic inputs using Sigmoid functions (Equation (3)) to emulate excitatory/inhibitory synapses. The parameter $x_{i,j}$ symbolizes the external signal received by the *j*th synapse on the *i*th branch. The constants $\omega_{i,j}$ and $\theta_{i,j}$ are sets of parameters specific to each synapse. By adjusting these parameters, the model can emulate the characteristics and functionalities of both excitatory and inhibitory synapses. Excitatory synapses $\left(0<\theta_{i,j}\leq \omega_{i,j}\right)$ and inhibitory synapses $\left(\omega_{i,j}<\theta_{i,j}<0\right)$ are modeled with distinct input–output relationships. In this model, BCs form excitatory connection with GCs while HCs form inhibitory connection with GC. Finally, GCs combine synaptic outputs from BCs and GCs via AND logic as shown in Equation (4):

(3) $$S_{i,j}=\frac{1}{1+e^{-k(\omega_{i,j}x_{i,j}-\theta_{i,j})}}$$

(4) $$Output=Output_{BC}\cdot \bar{Output_{HC}}$$

A previous study has presented a schematic representation of eight kinds of LMDNs, each corresponding to a distinct direction of motion. Figure 1B shows that the structure of the LMDN detects upward motion, the orange area represents the central pixel before the movement, while the blue area indicates its subsequent position after movement. The activation of the LMDN will occur only in response to motion along its preferred direction; in the example in Figure 1B, it only responds to upward motion.

### 2.2. GMM-Based Unsupervised AVS

The Gaussian Mixture Model (GMM) is an unsupervised learning algorithm designed to model complex data distributions by representing them as a mixture of multiple Gaussian components [36]. This method effectively captures underlying patterns in high-dimensional input data, making it a valuable tool for clustering and classification tasks. In biological systems, similar clustering mechanisms are believed to contribute to sensory processing, particularly in the organization of receptive fields in the visual cortex.

In the visual system, neurons in the primary visual cortex (V1) exhibit topographically organized receptive fields, meaning that nearby neurons respond to similar visual stimuli, a phenomenon observed in retinotopic maps [2,26]. Likewise, Gaussian Mixture Models (GMMs) categorize neurons with analogous response properties into distinct clusters, facilitating an efficient representation of motion information. Studies have shown that clustering mechanisms akin to GMMs play a crucial role in neural coding and perceptual organization [37,38]. This parallel between biological neural organization and GMM-based clustering has led to the adoption of GMMs as computational models for motion perception and feature integration in artificial vision systems [45,46].

**Figure 2:**
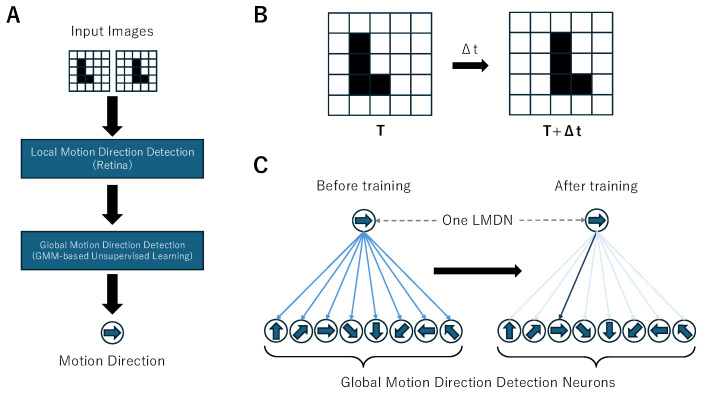
(**A**) Structure of GMM-based Unsupervised AVS. (**B**) An example of the input data. The input data comprise an image of an object before moving and an image of the object after moving, with a time difference of Δt between the two images. (**C**) A schematic diagram of the connection state between one LMDN and global motion direction-detecting neurons before and after training. Before training, the connections between the LMDN and global motion direction-detecting neurons are random. However, after training, only the connections between the most similar neurons are strengthened, while the others are weakened.

In this study, we combined the AVS for motion direction detection with the GMM to construct a GMM-based Unsupervised AVS for motion direction detection. This approach enables the formation of a global motion direction-detecting system through repeated exposure to motion stimuli, providing a more adaptive and biologically plausible method for motion detection in artificial visual systems. Figure 2A illustrates the overall framework of the model. The general structure of this model is similar to that of our previous work, as both consist of a local motion direction detection layer and a global motion direction detection layer. The primary difference lies in the output format of the local motion direction detection layer and the structure of the global motion direction detection layer.

The local motion direction detection layer retains the same mechanism as in our previous models, with a modified output format. The input format also remains consistent with prior models, comprising two consecutive image frames representing temporally adjacent visual stimuli. In previous studies, the identity of each local motion direction-detecting neuron was unimportant; we only needed to focus on binary values indicating whether the neurons were activated. In GMM-based Unsupervised AVS, we use a (8,2) matrix *I* as the output for each local motion direction-detecting neuron, in order to output the identity of each neuron. The eight dimensions correspond to the eight motion directions. We define the direction corresponding to the first dimension as rightward (0 degree), and as the dimensions increase, the corresponding direction rotates counterclockwise by 45 degrees. When a neuron is activated, it outputs a matrix where the dimension corresponding to the detected motion direction contains the index of the neuron’s position, while all other positions remain zero. If a neuron is not activated, it does not produce any output. The two components of index *I*, $I_{x}$ and $I_{y}$, are calculated using the following equations:

(5) $$I_{x}=a+h$$

(6) $$I_{y}=a+w$$

In the equations, to prevent the *I* from being entirely zero, we introduce a constant offset *a*. In this study, we set a=16. The variables *h* and *w* represent the neuron’s horizontal and vertical coordinates, respectively. Since we use 32 × 32 images as input, the maximum values of *h* and *w* are 31. The matrix of index *I* is shown below:

$$\left[\begin{array}{cc} I_{1x} & I_{1y}\\ I_{2x} & I_{2y}\\ I_{3x} & I_{3y}\\ I_{4x} & I_{4y}\\ I_{5x} & I_{5y}\\ I_{6x} & I_{6y}\\ I_{7x} & I_{7y}\\ I_{8x} & I_{8y} \end{array}\right]$$

In this matrix, each row represents a motion direction. The first column indicates the neuron’s x-coordinate, and the second column indicates the y-coordinate. For each neuron, only one row contains nonzero values. For example, the output matrix of a neuron that detects rightward motion is as follows:

$$\left[\begin{array}{cc} I_{1x} & I_{1y}\\ 0 & 0\\ 0 & 0\\ 0 & 0\\ 0 & 0\\ 0 & 0\\ 0 & 0\\ 0 & 0 \end{array}\right]$$

Before passing *I* to the GMM, we normalize and flatten it into a one-dimensional vector, as shown below:

$$\left[\begin{array}{ccccccccc} I_{1x}^{\prime } & I_{1y}^{\prime } & I_{2x}^{\prime } & I_{2y}^{\prime } & I_{3x}^{\prime } & I_{3y}^{\prime } & \ldots \ldots & I_{8x}^{\prime } & I_{8y}^{\prime } \end{array}\right]$$

Then, we pass these indexed data into the GMM-based global motion direction detection layer. In this layer, we apply the Gaussian Mixture Model (GMM) principle to cluster LMDNs corresponding to each motion direction. According to the GMM mechanism, each data point is assumed to be generated from a mixture of multiple Gaussian distributions. The probability of a data point belonging to a particular Gaussian component is determined based on the Expectation-Maximization (EM) algorithm.

The responsibility of each Gaussian component for a given input vector *I* is computed using the following equation:

(7) $$R_{ik}=\frac{\pi_{k}N\left(I|\mu_{k},\Sigma_{k}\right)}{\sum_{j=1}^{K}\pi_{j}N\left(I|\mu_{j},\Sigma_{j}\right)}$$

where $R_{ik}$ represents the responsibility of the *k*-th Gaussian component for the input vector *I*, $\pi_{k}$ is the mixture weight, $\mu_{k}$ is the mean, and $\Sigma_{k}$ is the covariance matrix of the *k*th Gaussian distribution.

During the training process, once the responsibilities are computed, the parameters of the Gaussian components are updated iteratively using the Expectation-Maximization (EM) algorithm. The parameter update process in the GMM model is governed by the following equations:

1. Mean Update Equation:
  The mean of each Gaussian component is updated based on the weighted sum of all input vectors, where $N_{k}$ represents the effective number of data points assigned to the *k*th Gaussian component. This concept reflects the soft clustering nature of the GMM, where data points are not assigned to a single cluster but distributed across components with associated probabilities.
  (8) $$\mu_{k}^{new}=\frac{1}{N_{k}}\sum_{i=1}^{N}R_{ik}I_{i}$$
2. Covariance Matrix Update:
  The covariance matrix of each Gaussian component is updated based on the weighted sum of the squared differences between the input vectors and the updated mean.
  (9) $$\Sigma_{k}^{new}=\frac{1}{N_{k}}\sum_{i=1}^{N}R_{ik}\left(I_{i}-\mu_{k}^{new}\right){\left(I_{i}-\mu_{k}^{new}\right)}^{T}$$
3. Mixing Coefficient Update:
  The mixing coefficient, which determines the proportion of data points assigned to each Gaussian component, is updated as follows:
  (10) $$\pi_{k}^{new}=\frac{N_{k}}{N}$$
  where *N* is the total number of data points.
4. Log-Likelihood Computation:
  To assess the convergence of the EM algorithm, the log-likelihood of the observed data is computed at each iteration. The algorithm iterates until the log-likelihood converges to a stable value.
  (11) $$\log L=\sum_{i=1}^{N}\log\sum_{k=1}^{K}\pi_{k}N\left(I_{i}|\mu_{k},\Sigma_{k}\right)$$

Once the GMM training is complete, we can use it to classify the LMDNs. For an LMDN, we calculate its responsibility for each Gaussian component using Equation (7). LMDNs are then assigned to the component with the highest responsibility. This equation is used to perform a hard assignment of LMDNs to a specific Gaussian component after the GMM has been trained.

(12) $$k^{*}=\arg\max_{k}\gamma_{ik}$$

The labels assigned to LMDNs by the GMM can be seen as the connection between the LMDNs and the global motion direction-detecting layer, meaning that each LMDN is linked to a global motion direction-detecting neuron corresponding to its label. Before training the GMM, these labels are randomly assigned, meaning that the connections between LMDN and the global motion direction-detecting layer are random. However, after training, all LMDNs that detect motion in the same direction will be connected to the same global motion direction-detecting neuron. Figure 2C illustrates the schematic diagram of the connection between one LMDN and global motion direction-detecting neurons before and after training. The objects at the top represent an LMDN, while those at the bottom represent the global motion direction detection layer. The lines in the middle indicate the connections between the two layers. Before training, the connections between LMDNs and global motion direction-detecting neurons are random, meaning that each LMDN has an equal probability of being assigned to any direction. However, after training, only the connections between the neurons with the most similar preferred direction are strengthened, while the others are weakened. As a result, strong connections are preserved only among neurons tuned to the same motion direction, indicating that LMDNs detecting the same direction are most likely to be grouped together.

## 3. Results

In the first experiment, we assume an ideal case that after birth, a biological system has sufficient time to observe motion in all directions without perceptual impairments. We assume that the resolution of the retina is 32 × 32, with eight LMDNs detecting different motion directions beneath each pixel. As a result, the 32 × 32 retinal array consists of 8192 individual neurons. Therefore, the training dataset consists of the outputs of all LMDNs across all pixels, forming a comprehensive dataset with a shape of (8192, 8, 2). We initialized the GMM with eight components without performing training. The initial means of the components are manually set using random values rather than being inferred from the data. We generated a random number, 8595, as the seed for this study. The covariance matrices are initialized as identity matrices, ensuring an isotropic variance structure. The component weights are uniformly distributed, assigning equal prior probability to each component. Finally, the Cholesky decomposition of the precision matrices is computed based on the inverse of the covariance matrices.

**Figure 3:**
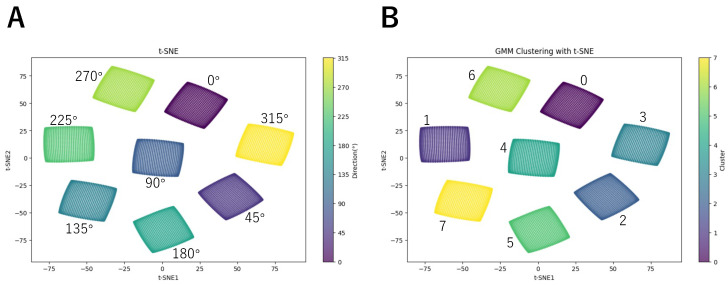
(**A**) Results of t-SNE dimensionality reduction with the true labels. (**B**) The t-SNE dimensionality reduction results using the labels assigned by the trained GMM.

To better visualize the classification results, we used t-SNE to reduce the dimensionality of the LMDN outputs and visualize them. The reason for choosing t-SNE is that it can map LMDNs detecting the same motion direction to nearby regions after dimensionality reduction, forming distinct clusters or ’islands’ for each direction. Figure 3A illustrates the results of t-SNE dimensionality reduction. We labeled the data using the original tags. The results show that LMDNs detecting different directions are grouped into separate islands, allowing us to determine the true motion direction represented by each cluster.

**Figure 4:**
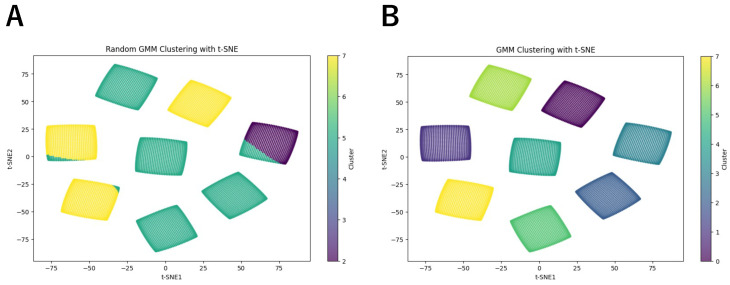
(**A**) Results of t-SNE dimensionality before training GMM. (**B**) The t-SNE dimensionality reduction results using the labels assigned by the trained GMM.

Next, we classified the LMDNs using GMM. We first performed classification without training the GMM, generating initial labels. The second row of Table 1 shows the number of elements assigned to each label before training, while Figure 4A presents the t-SNE visualization with these untrained labels. The results indicate that the untrained GMM failed to correctly classify LMDNs by direction. We then trained the GMM using the full dataset with a shape of (8192, 8, 2). By default, the GMM runs up to 100 iterations until convergence or until reaching the max_iter limit. In this experiment, the training process converged within two epochs. The third row of Table 1 shows the number of elements assigned to each label after training, while Figure 4B presents the t-SNE visualization with labels from the trained GMM. The results demonstrate that the elements were correctly assigned, with only one label appearing in each island. Since GMM-based classification does not inherently determine the angle associated with each label, we referenced the t-SNE visualization in Figure 3 to identify the corresponding angle for each island. This allows us to determine the directional angle for each label. Table 2 presents the mapping between labels, angles, and motion directions.

**Figure 5:**
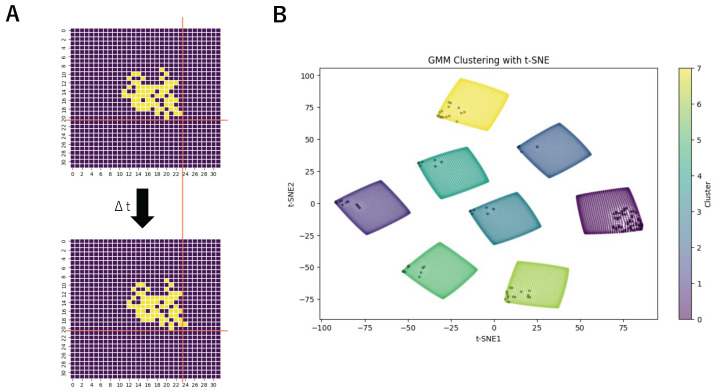
(**A**) Two input images. The object moved toward the right for one pixel. (**B**) The distribution map of activated neurons after visualizing with t-SNE.

Subsequently, we embedded the trained GMM parameters into the AVS to construct a GMM-based Unsupervised AVS. To verify whether the model was successfully trained, we randomly generated a pair of images containing a 64-pixel object moving to the right for testing. When these images were input into the GMM-based Unsupervised AVS, the local motion direction detection layer first processed them, activating several LMDNs, which then sent signals to the GMM-based global motion detection neuron layer. These signals were classified by the GMM using assigned labels. By applying t-SNE for dimensionality reduction, we obtained an activation map, as shown in Figure 5. Due to the intrinsic requirements of t-SNE, we visualized the activated LMDNs by concatenating them with the previously used full dataset. This resulted in a shift in island positions compared to earlier experiments. However, since the GMM-assigned labels remained unchanged, the direction corresponding to each label remained the same as in Figure 3. In Figure 5, we marked the newly activated neurons with black circles.

From Figure 5B, we can observe that signals corresponding to different motion directions have clustered into distinct regions. All activated neurons are located within the islands corresponding to their detected motion directions. Additionally, label 0, which represents neurons detecting 0-degree (rightward) motion, was activated the most, aligning with the motion direction of the object in the image. We counted the number of activated neurons in each region and found that these numbers match the counts of activated local motion detection neurons for the corresponding directions, as shown in Table 3. As a result, the GMM layer successfully classified LMDNs detecting different directions, allowing us to determine the global motion direction by comparing the activation counts across directions.

**Figure 6:**
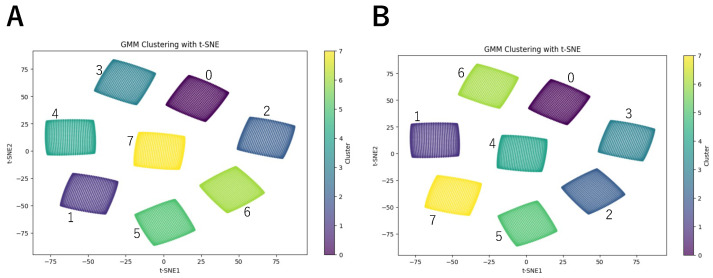
(**A**) The t-SNE dimensionality reduction results using the labels assigned by the GMM trained on 10% of the full dataset. (**B**) The t-SNE dimensionality reduction results using the labels assigned by the GMM trained on the full dataset.

To further validate the feasibility of our model, we trained it using only 10% of the complete training dataset. We randomly chose 10% of data from the complete training dataset and trained the model. The results are shown in Figure 6. Even with a smaller training set, the model still successfully separated the regions corresponding to different motion directions. The key difference compared to the fully trained model is that the angle associated with each label has changed. This indicates that after each training session, the model must verify the correspondence between labels and motion directions before it can be used.

Finally, we conducted an accuracy experiment. We embedded the GMM trained on the full dataset into the AVS, forming the GMM-based Unsupervised AVS. In this system, when an image pair is input, certain LMDNs are activated. The signals from these activated LMDNs are then passed to the GMM layer, where they are automatically classified according to their corresponding motion directions. The model then outputs the number of activated LMDNs for each direction. The final motion direction of the object is determined based on the direction associated with the highest number of activated LMDNs.

**Figure 7:**
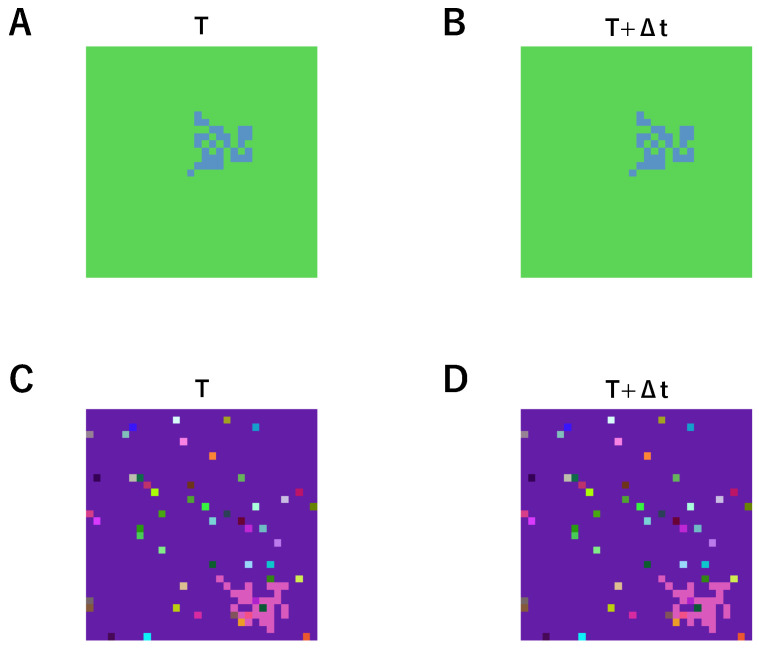
(**A**,**B**) Examples of Dataset-C. (**C**,**D**) Examples of NoiseType1.

We utilized Dataset-C and NoiseType1 from an earlier study by Hua et al. [35] to conduct our experiments. Examples from each of the two datasets are shown separately in Figure 7. Dataset-C is a color image dataset in which both the object and background in each sample are assigned randomly selected, uniform colors that remain constant throughout the object’s motion. NoiseType1 introduces visual noise by injecting randomly colored pixels at random spatial locations; these noise pixels remain static and do not move with the object. Table 4 presents the test results. The results show that our GMM-based Unsupervised AVS produced the same outcomes as the AVS developed by Hua et al. [35], demonstrating that a GMM-based global layer can perform the same function as the global layer in previous studies.

## 4. Summary

In this paper, we combined the motion direction-detecting AVS with a Gaussian Mixture Model (GMM) to construct an unsupervised motion direction-detecting model, simulating the effects of early visual experience on the development of the visual system. The model consists of two main components:

1. A local motion direction detection layer, which corresponds to the retina and retains the previously established structure and mechanisms.
2. A global motion direction detection layer, which was redesigned from a simple summation-based approach to a GMM-based unsupervised learning mechanism.

We used the GMM training process to simulate how early visual experience shapes the development of the motion direction recognition in visual systems. When trained on a dataset that simulated a normal environment (8-direction stimuli), the model produced results consistent with prior summation-based models, confirming its validity in motion detection. Additionally, our model introduces a new approach for detecting macroscopic motion direction. We apply unsupervised learning to classify local motion direction-detecting neurons, clustering neurons that detect the same direction to form macroscopic motion direction detection neurons. The outputs of these macroscopic neurons are proportional to the input signals they receive. Ultimately, the neuron with the highest output indicates the macroscopic motion direction of the object. This study not only proposes a new motion direction-detecting model but also introduces a theoretical framework for understanding how early visual experience influences visual system development.

## 5. Discussion

In this study, our primary goal was to validate the model’s conceptual soundness and biological plausibility, rather than to optimize for performance on large-scale datasets. Although the proposed LMDN-GMM framework demonstrates the potential of biologically inspired unsupervised learning for motion direction detection, we acknowledge that the current output structure of the LMDN layer—specifically, the use of an (8,2) matrix per neuron—is not necessarily the most efficient or optimal choice for representing motion features. This format was originally selected to balance biological relevance and computational simplicity; however, it may impose limitations in terms of scalability and performance.

In future work, we aim to systematically investigate alternative model architectures and data representations that enhance computational efficiency while maintaining consistency with biological principles. This includes experimenting with lower-dimensional outputs, dynamic connection strategies, and adaptive data encoding schemes. Additionally, further investigation is needed to determine the most suitable forms of unsupervised learning for capturing motion direction in a robust and scalable manner.

In conclusion, while our current implementation provides a foundational step toward biologically inspired motion processing, we recognize its limitations and are committed to refining both the model and its data structures to achieve better efficiency and accuracy.

## Figures and Tables

**Table 1:** Labels and number of components.

Label	0	1	2	3	4	5	6	7
Random	0	0	788	0	0	4450	0	2954
Trained	1024	1024	1024	1024	1024	1024	1024	1024

**Table 2:** Relations between labels and directions.

**Label**	**0**	**1**	**2**	**3**
**Direction**	Rightward	Left-Lower	Right-Upper	Right-Lower
**Angle**	0°	225°	45°	315°
**Label**	**4**	**5**	**6**	**7**
**Direction**	Upward	Leftward	Downward	Left-Upper
**Angle**	90°	180°	270°	135°

**Table 3:** Activated neurons and directions.

**Label**	**0**	**1**	**2**	**3**
**Direction**	**Rightward**	Left-Lower	Right-Upper	Right-Lower
**Activations**	**56**	11	5	7
**Label**	**4**	**5**	**6**	**7**
**Direction**	Upward	Leftward	Downward	Left-Upper
**Activations**	7	11	16	17

**Table 4:** Result of accuracy test.

Size/Noise	0%	1%	5%	10%
1	97.50%	97.63%	94.75%	92.25%
2	98.75%	97.88%	97.00%	93.63%
4	99.13%	99.75%	98.50%	97.38%
8	100%	100%	100%	99.38%
16	100%	100%	100%	100%
32	100%	100%	100%	100%
64	100%	100%	100%	100%
128	100%	100%	100%	100%
256	100%	100%	100%	100%
512	100%	100%	100%	100%

## Data Availability

The data that support the findings of this study are available from the corresponding author upon reasonable request.
